# Supplementary figures and images for: A neurogenic signature involving monoamine Oxidase-A controls human thermogenic adipose tissue development
Source: eLife. 2022 Sep 15;11:e78945. doi: 10.7554/eLife.78945 (PMC9519151; doi:10.7554/eLife.78945)

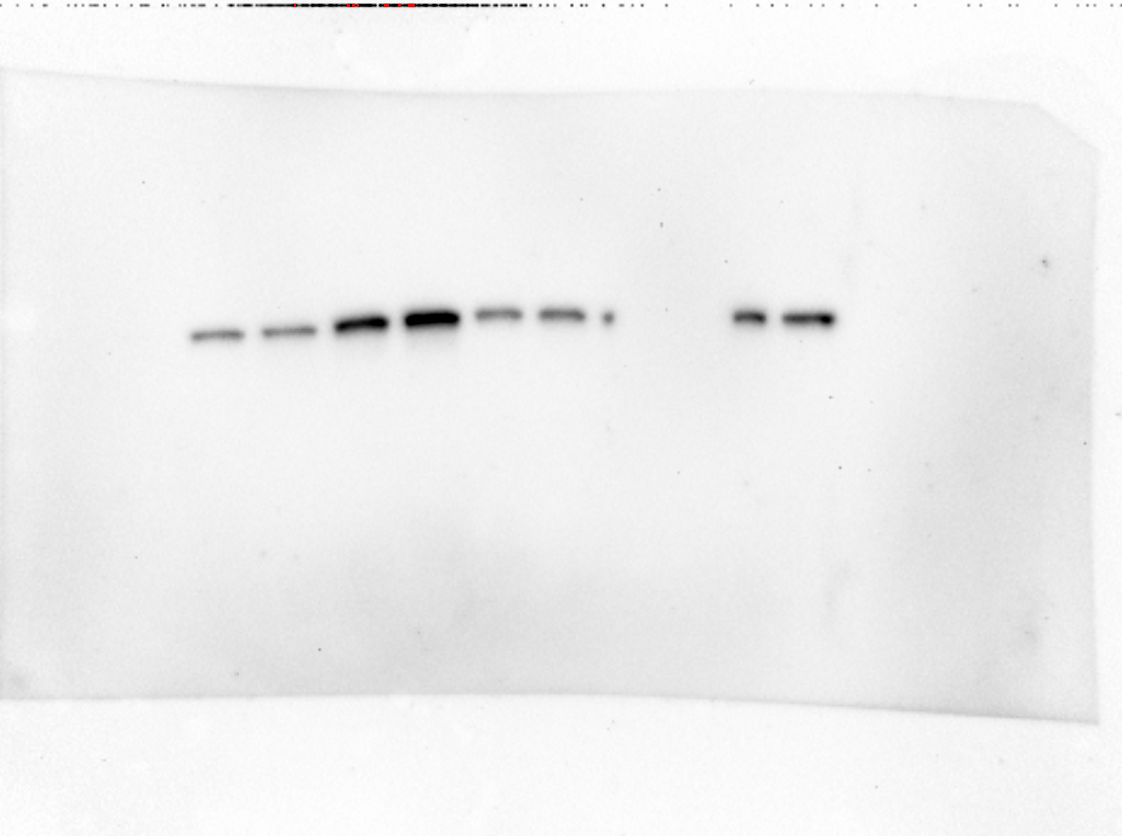

Supplement: Figure 6—source data 1. [file elife-78945-fig6-data1.zip › Figure_6_SOURCE_DATA/MAOA_Hu63_60secExp_solivanj 2021-09-28_13h52m39s.tif]

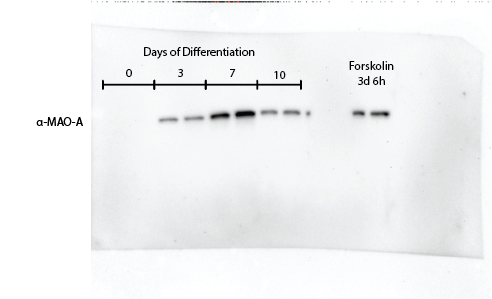

Supplement: Figure 6—source data 1. [file elife-78945-fig6-data1.zip › Figure_6_SOURCE_DATA/FIGURE_6_SOURCE_DATA_MAOA.png]
